# Supplementary material for: Superior thermoelasticity and shape-memory nanopores in a porous supramolecular organic framework
Source: Nat Commun. 2016 May 11;7:11564. doi: 10.1038/ncomms11564 (PMC4865851; doi:10.1038/ncomms11564)
Supplement: Supplementary Information — Supplementary Figures 1-14, Supplementary Tables 1-7 and Supplementary References. [file ncomms11564-s1.pdf]

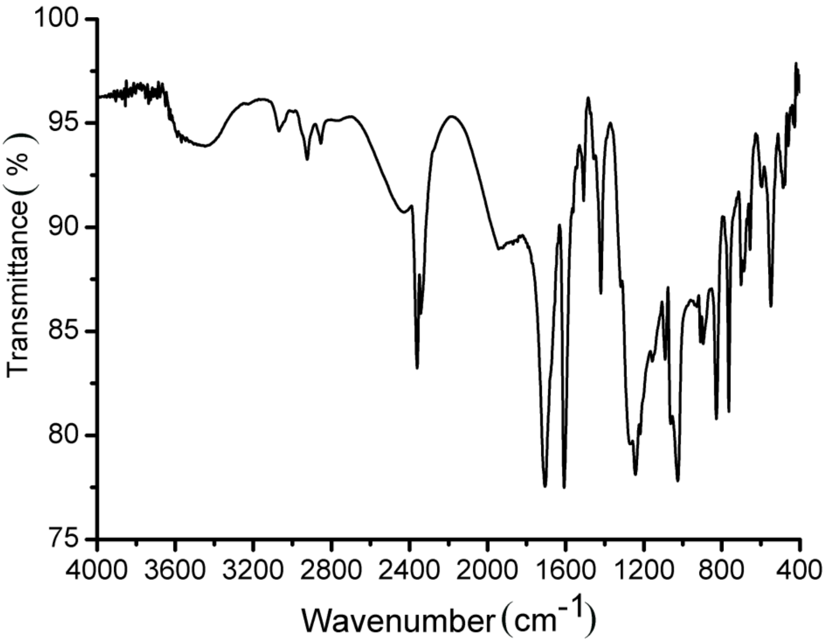

Supplementary Figure 1. IR spectrum for compound **1o**·0.33DMF.

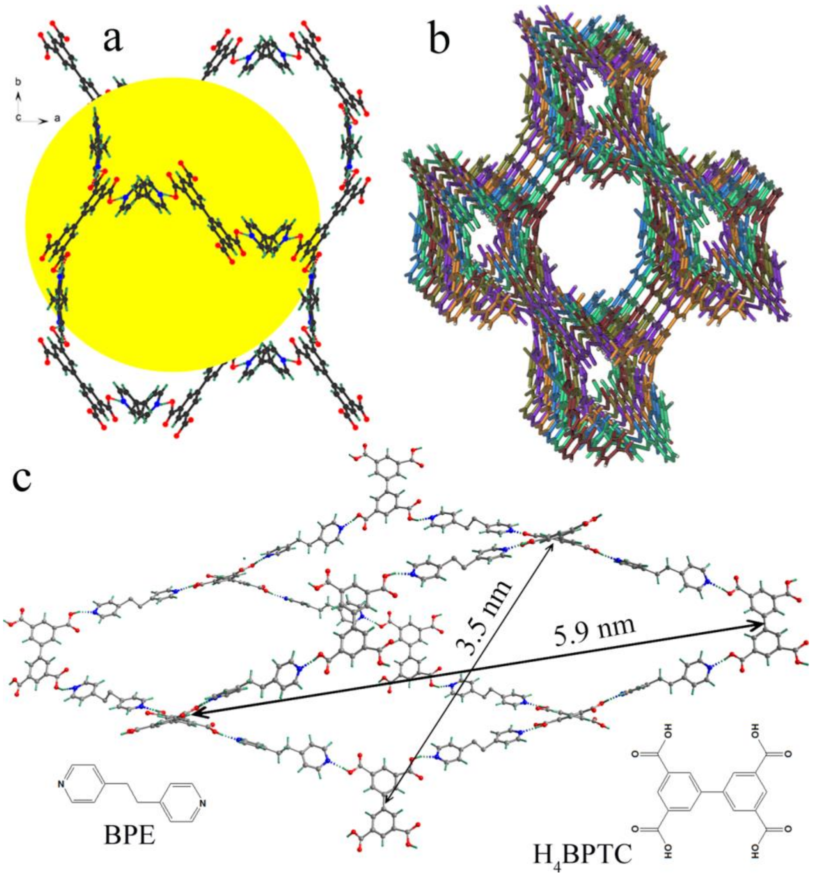

Supplementary Figure 2. **a**, The colossal adamantine cage assembled by four helicates in compound **1o**·0.33DMF; **b**, six single ***dia*** frameworks interpenetrate in parallel along the *c*-axis, forming the sextuple interpenetrated ***dia*** framework; **c**, the colossal adamantine cage showing huge windows (5.9 × 3.5 nm^2), and molecular structures of BPE and H_4BPTC.

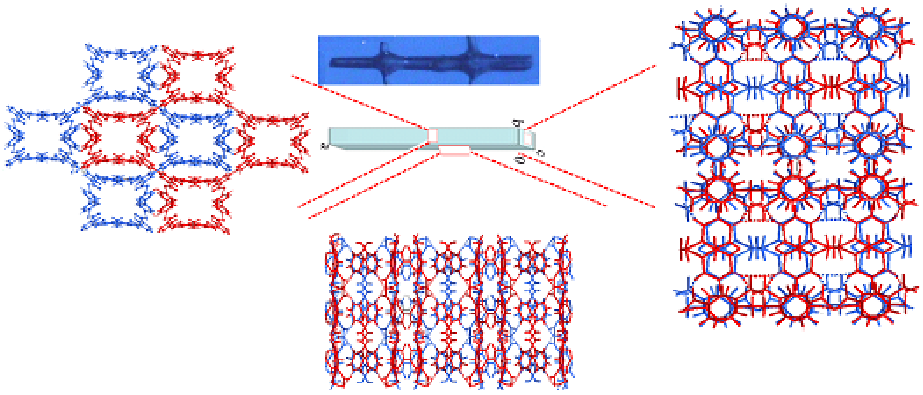

Supplementary Figure 3. Panoramic view of the crystal structure for compound **1o**·0.33DMF.

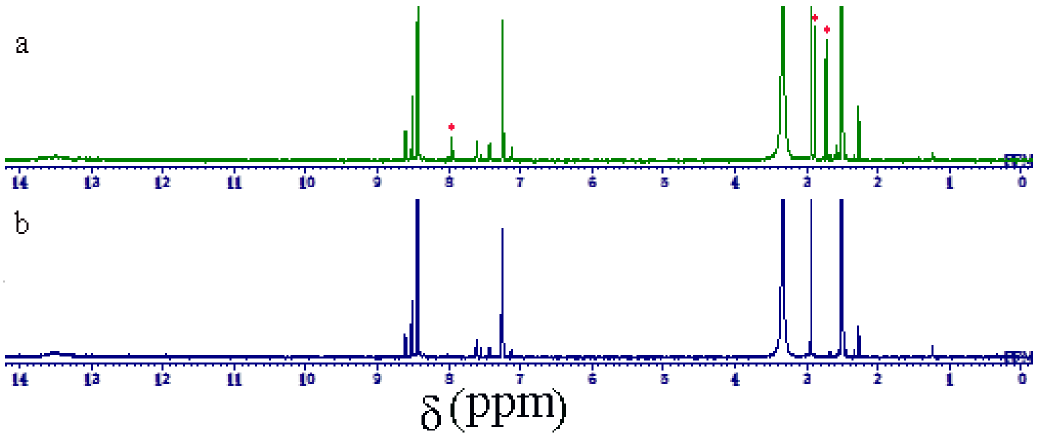

Supplementary Figure 4. a, ^{1}H NMR (400 MHz, DMSO-d_6) for as-prepared compound **1o**·0.33DMF; **b**, ^{1}H NMR for compound **1o** generated by desolvation under vacuum at 60 °C. The chemical shifts of DMF, δ = 2.73, 2.89 and 7.95 ppm, are quite apparent for the as-prepared compound **1o**·0.33DMF, whereas they are almost invisible for compound **1o**. This implies that DMF was completely removed in compound **1o**.

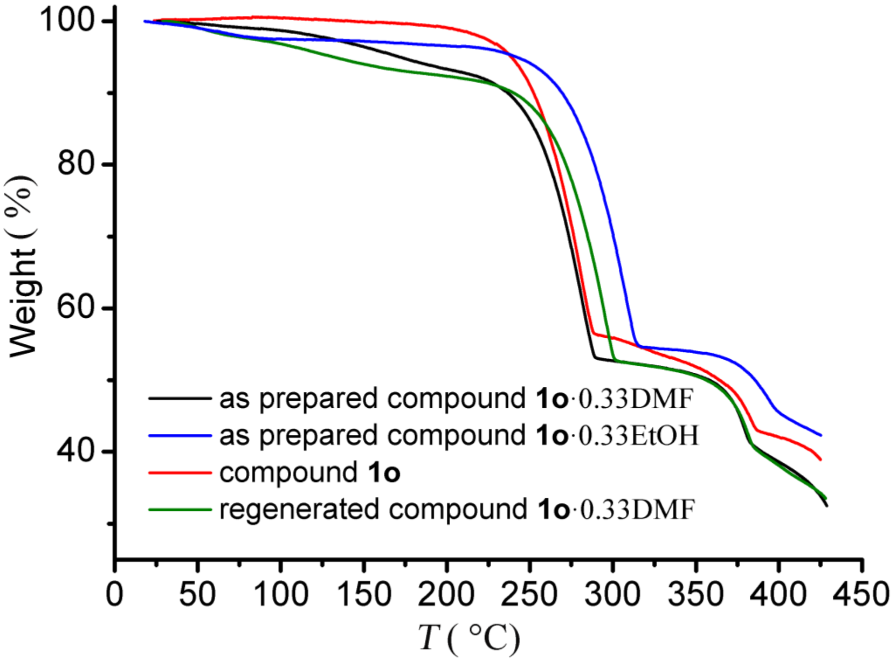

Supplementary Figure 5. TGA traces for compound **1o**·0.33DMF, compound **1o**·0.33EtOH, compound **1o**, and regenerated compound **1o**·0.33DMF.

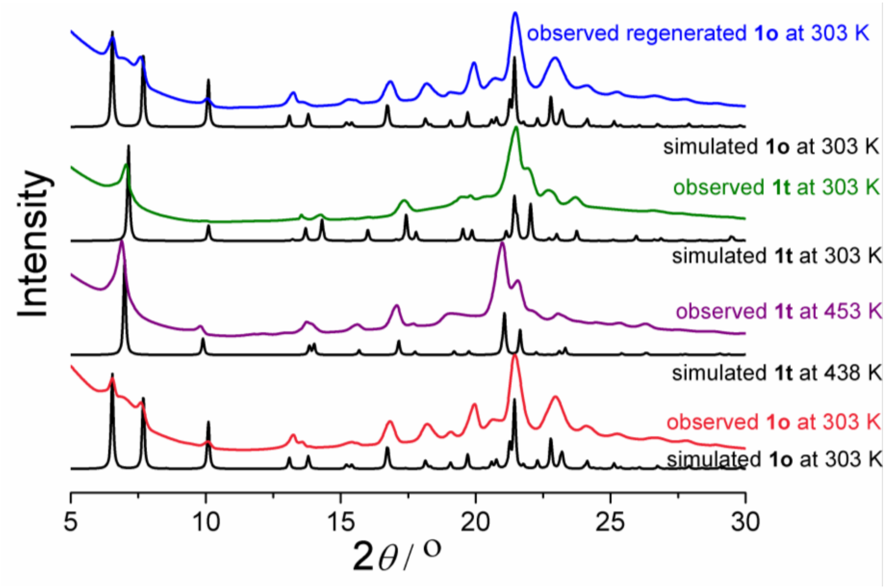

Supplementary Figure 6. PXRD patterns of compound **1o**, compound **1t**, and regenerated compound **1o** compared with patterns simulated from single-crystal structures.

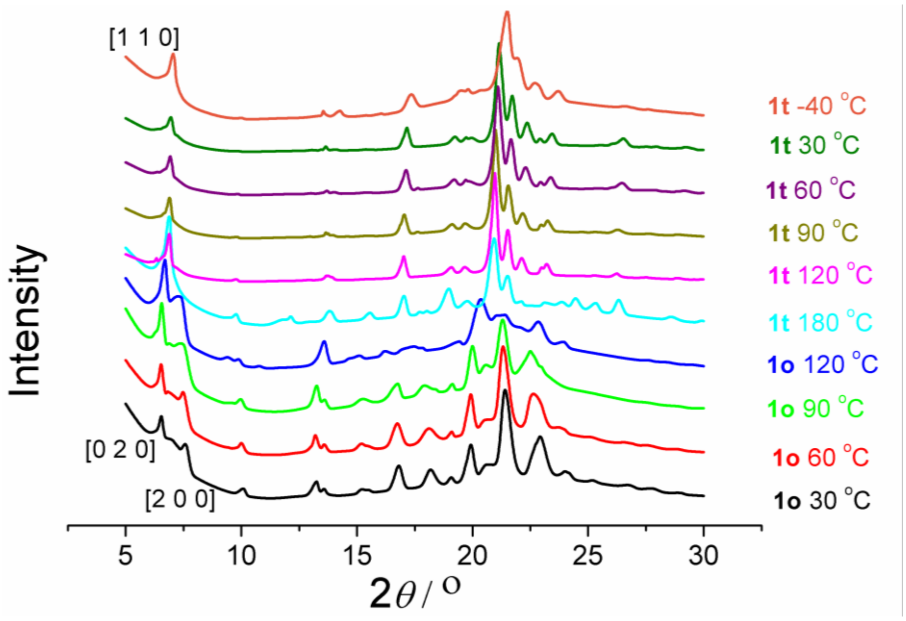

Supplementary Figure 7. PXRD pattern evolution of desolvated **1o**·0.33 DMF, showing the irreversible structural transition from compound **1o** to compound **1t**.

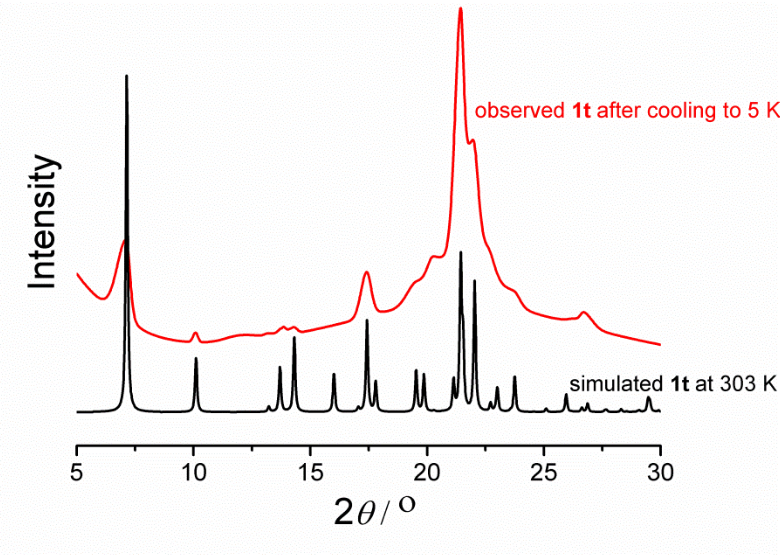

Supplementary Figure 8. PXRD pattern of compound **1t** pre-cooled at 5 K, the experimental pattern was measured at 303 K.

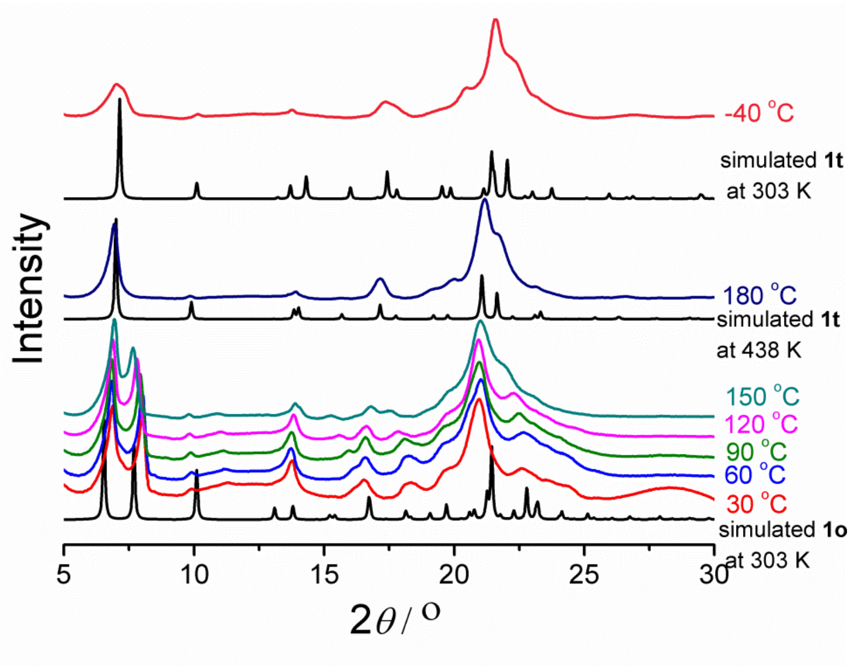

Supplementary Figure 9. PXRD pattern evolution of desolvated compound **1o**·0.33EtOH, showing the thermally irreversible structural transition from compound **1o** to compound **1t**.

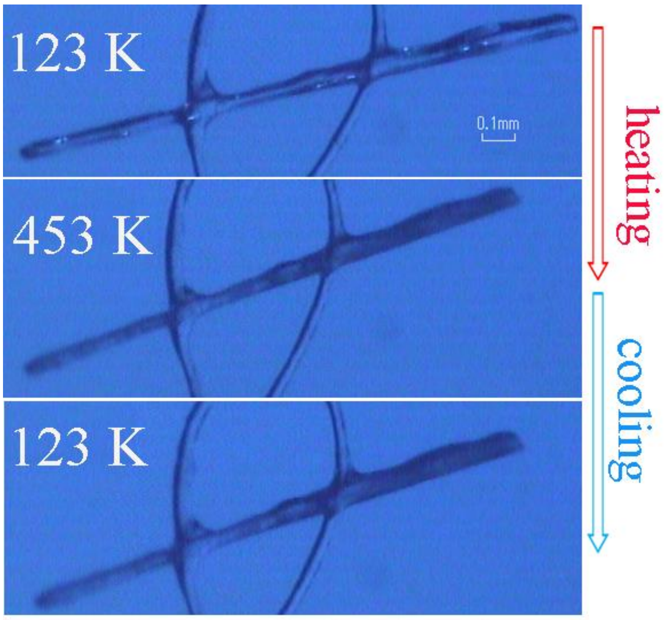

Supplementary Figure 10. Single-crystal photographs showing the thermally irreversible phase transition from compound **1o** to compound **1t**. A needle–shaped single crystal is mounted on a nylon loop, photographs of which are taken with a camera equipped on a Rigaku CCD diffractometer.

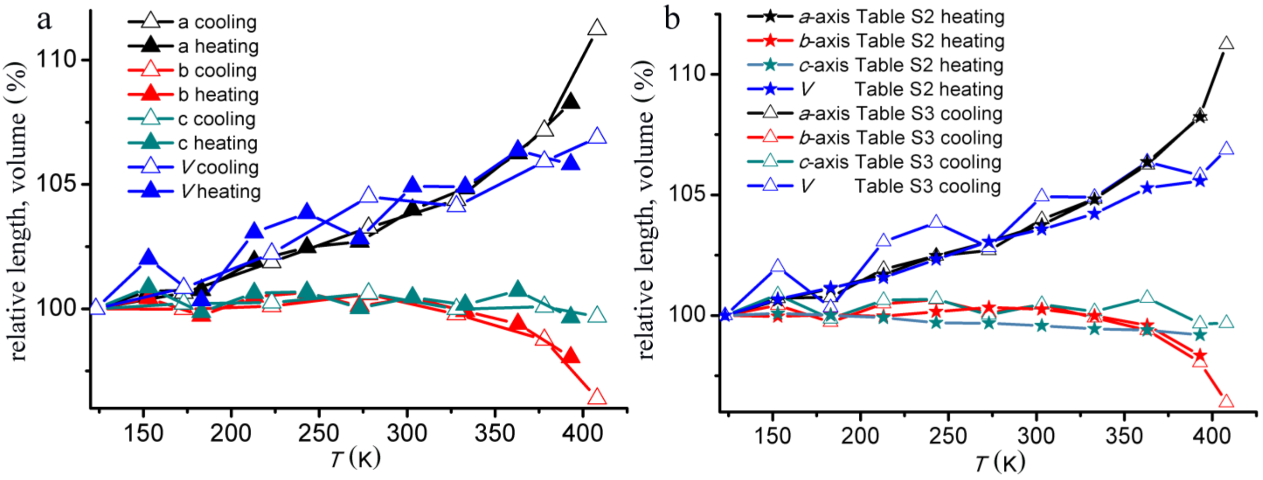

Supplementary Figure 11. **a**, Temperature-dependent unit cell parameters for compound **1o** showing superior thermoelasticity. All parameters are normalized by those determined at 123 K, and the cell parameters are summarized in Supplementary Table 3. **b**, Plots showing that the temperature dependent change of the *a*, *b*, *c* parameters, and *V* for two different crystals is consistent with each other.

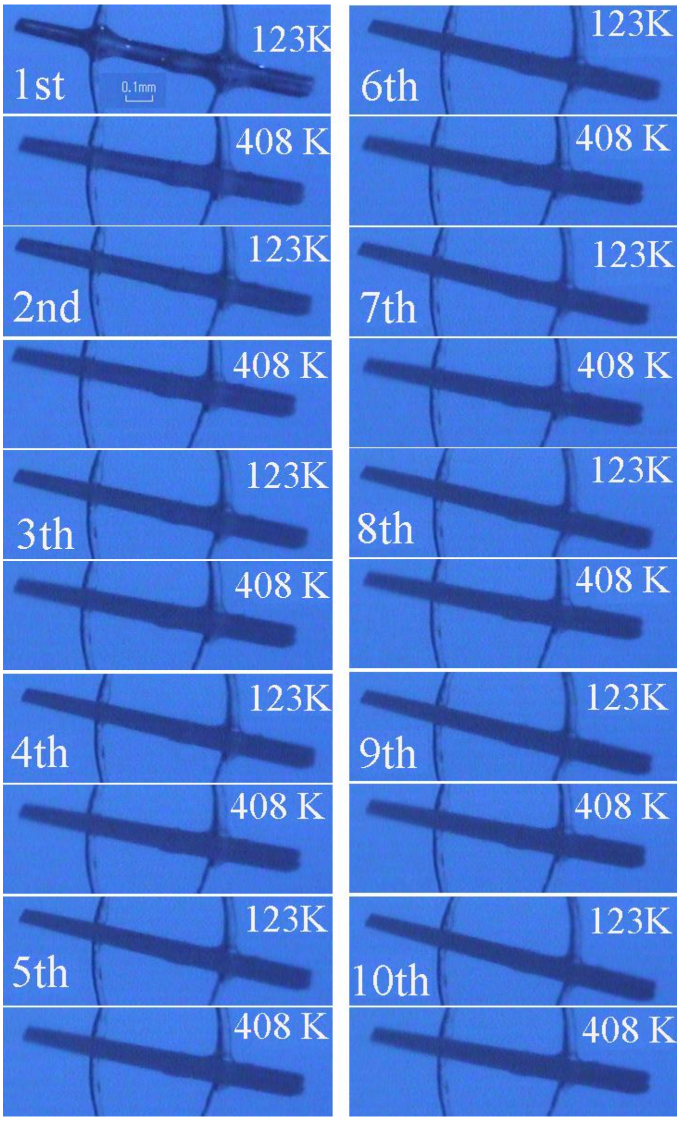

Supplementary Figure 12. Photographs of a needle–shaped single-crystal showing superior thermoelasticity for compound **1o**. The photographs of the crystal mounted on a nylon loop are taken with a camera equipped on a Rigaku CCD diffractometer.

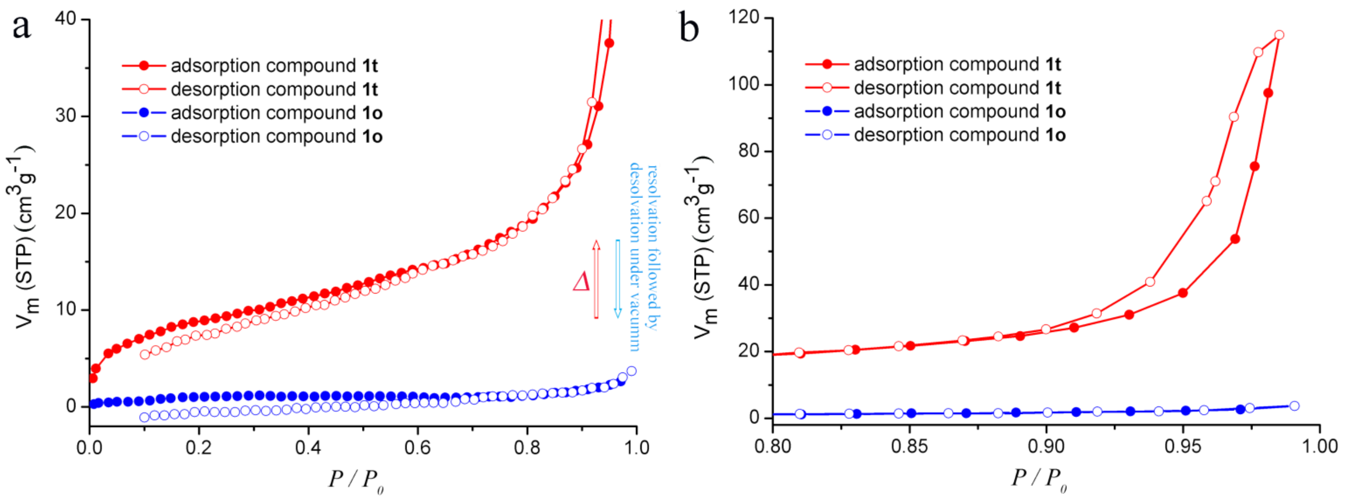

Supplementary Figure 13. a, N_2 sorption isotherms for compound **1o** and **1t** at 77 K, and **b**, the isotherms at high relative pressure.

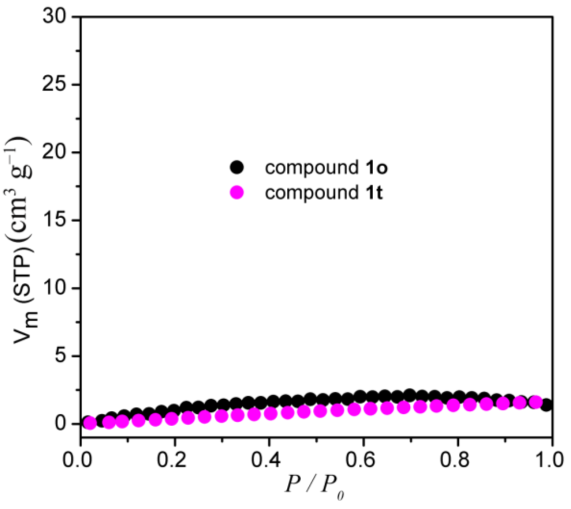

Supplementary Figure 14. CO_2 adsorption isotherms for compound **1o** and **1t** at 196 K.

Supplementary Table 1. Crystallographic data for compound **1o**·0.33DMF.

Compound	Compound **1o**·0.33DMF
Formula	$C_{20}H_{17}N_2O_4$
Fw	349.35
Crystal size(mm)	0.12 × 0.06 × 0.06
Space group	Ccca
a = (Å)	22.313(4)
b = (Å)	26.967(5)
c = (Å)	12.873(3)
V (Å^3)	7746(3)
Z	16
D_c(g/cm^3)	1.198
T(K)	123
Rint	0.0988
Parameters	271
F(000)	2928
data collected	24734
unique data	3448
R_1, wR(I > 2σ(I))[a]	0.1373, 0.3141
R_1, wR(all data)[b]	0.1737, 0.3390
Goodness-of-fit-on F^2	1.074
CCDC[#]	1423054

[a] $R = \sum||F_0|-|F_c||)/\sum|F_0|$

[b] $wR = |\sum w(F_0-F_c)^2/\sum w(F_0^2)^2|^{1/2}$

Supplementary Table 2. Crystallographic data for compound **1o** upon heating from 123 to 438 K and then cooling back to 123 K.

T(K)	123	153	183	213	243
Formula	$C_{20}H_{17}N_2O_4$	$C_{20}H_{17}N_2O_4$	$C_{20}H_{17}N_2O_4$	$C_{20}H_{17}N_2O_4$	$C_{20}H_{17}N_2O_4$
Fw	349.35	349.35	349.35	349.35	349.35
Crystal size(mm)	0.14 × 0.09 × 0.09	0.14 × 0.09 × 0.09	0.14 × 0.09 × 0.09	0.14 × 0.09 × 0.09	0.14 × 0.09 × 0.09
Space group	Ccca	Ccca	Ccca	Ccca	Ccca
a = (Å)	22.143(5)	22.283(5)	22.390(5)	22.519(5)	22.691(5)
b = (Å)	26.947(6)	26.935(6)	26.954(6)	26.938(6)	26.989(6)
c (Å)	12.880(3)	12.891(3)	12.880(3)	12.867(3)	12.841(3)
V(Å^3)	7685(3)	7737(3)	7773(3)	7798(8)	7864(3)
Z	16	16	16	16	16
D_c(g/cm^3)	1.208	1.200	1.194	1.189	1.180
*R*int	0.0525	0.0542	0.0481	0.0796	0.0511
Parameters	271	271	271	271	271
F(000)	2928	2928	2928	2928	2928
data collected	30135	30548	30582	30712	30876
unique data	3487	3504	3516	3528	3555
R_1, wR(I > 2σ(I))[a]	0.1475, 0.3733	0.1406, 0.3611	0.1362, 0.3340	0.1430, 0.3729	0.1347, 0.3316
R_1, wR(all data)[b]	0.1628, 0.3849	0.1542, 0.3714	0.1499, 0.3442	0.1610, 0.3862	0.1542, 0.3453
Goodness-of-fit-on F^2	1.155	1.170	1.122	1.126	1.176
CCDC[#]	1423057	1423058	1423059	1423060	1423061

T(K)	273	303	333	363	393
Formula	$C_{20}H_{17}N_2O_4$	$C_{20}H_{17}N_2O_4$	$C_{20}H_{17}N_2O_4$	$C_{20}H_{17}N_2O_4$	$C_{20}H_{17}N_2O_4$
Fw	349.35	349.35	349.35	349.35	349.35
Crystal size(mm)	0.14 × 0.09 × 0.09	0.14 × 0.09 × 0.09	0.14 × 0.09 × 0.09	0.14 × 0.09 × 0.09	0.14 × 0.09 × 0.09
Space group	Ccca	Ccca	Ccca	Ccca	Ccca
a = (Å)	22.819(6)	22.975(5)	23.210(5)	23.552(6)	23.966(8)
b = (Å)	27.035(7)	27.014(6)	26.944(6)	26.838(7)	26.500(8)
c = (Å)	12.839(3)	12.824(3)	12.808(3)	12.801(3)	12.776(4)
V (Å^3)	7920(4)	7960(3)	8009(3)	8091(3)	8114(4)
Z	16	16	16	16	16
D_c(g/cm^3)	1.172	1.166	1.159	1.147	1.144
*R*int	0.0507	0.0523	0.0552	0.0565	0.0695
Parameters	271	271	271	271	271
F(000)	2928	2928	2928	2928	2928
data collected	30861	31177	31497	31821	31684
unique data	3578	3601	3631	3670	3670
R_1, wR(I > 2σ(I))[a]	0.1438, 0.3428	0.1384, 0. 3404	0.1385, 0.3228	0.1867, 0.4090	0.2317, 0.4529
R_1, wR(all data)[b]	0.1693, 0.3594	0.1690, 0.3604	0.1713, 0.3425	0.2216, 0.4323	0.2650, 0.4740
Goodness-of-fit-on F^2	1.181	1.205	1.182	1.213	1.278
CCDC[#]	1423062	1423063	1423064	1423065	1423066

T(K)	438	303*	123*		

Formula	$C_{20}H_{17}N_2O_4$	$C_{20}H_{17}N_2O_4$	$C_{20}H_{17}N_2O_4$
Fw	349.35	349.35	349.35
Crystal size(mm)	0.14 × 0.09 × 0.09	0.14 × 0.09 × 0.09	0.14 × 0.09 × 0.09
Space group	$P4_2/nbc$	$P4_2/nbc$	$P4_2/nbc$
a = (Å)	17.856(6)	17.491(9)	17.289(6)
b = (Å)	17.856(6)	17.491(9)	17.289(6)
c = (Å)	12.787(7)	12.915(7)	12.957(4)
V (Å^3)	4077(3)	3951(4)	3873(2)
Z	16	16	16
D_c(g/cm^3)	1.138	1.175	1.198
*R*int	0.0965	0.0945	0.0999
Parameters	120	119	119
F(000)	1464	1464	1464
data collected	14093	13652	13627
unique data	1831	1795	1744
R_1, *w*R(I > 2σ(I))[a]	0.1735, 0.4425	0.2921, 0.6352	0.2856, 0.5842
R_1, *w*R(all data)[b]	0.2123, 0.4742	0.3202, 0.6533	0.3197, 0.6084
Goodness-of-fit-on F^2	0.913	0.935	0.985
CCDC[#]	1423067	1423068	1423069

[a] $R = \sum||F_0|-|F_c||)/\sum|F_0|$

[b] $wR = |\sum w(F_0-F_c)^2/\sum w(F_0^2)^2|^{1/2}$

*The crystallographic data upon cooling back to 303 K and 123 K are very poor because of the deterioration of the crystal quality after heating at 438 K for 2 h for data collection..

Supplementary Table 3. Evolution of cell parameters for compound **1o** determined in a cooling and heating cycle from 408 to 123 K. The single crystal used for this measurement is different from that used for Table *S2*. Because the temperature of the single crystal for Table *S2* was increased above phase transition temperature (438 K), it irreversibly transitioned to the tetragonal phase and the quality of the crystal is decreased. Therefore, a new single crystal was used to show reversible change in the crystal *a*, *b*, *c* parameters, and *V* below the phase transition temperature. As shwon in Fig. *S9(b)*, the temperature dependent change in the *a*, *b*, *c* parameters, and *V* is consistent with each other.

T(K)	*a* = (Å)	*b* =(Å)	*c* = (Å)	*V* (Å^3)	
408	24.9286	26.2659	12.8684	8425.8695	cooling
378	24.0161	26.9108	12.9205	8350.4218	
328	23.3899	27.1903	12.9075	8208.8912	
278	23.1408	27.4100	12.9895	8239.1012	
223	22.8293	27.2762	12.9415	8058.6274	
173	22.5540	27.2458	12.9360	7949.1949	
123	22.4101	27.2503	12.9097	7883.7207	
153	22.5698	27.3647	13.0210	8041.9754	heating
183	22.5783	27.1746	12.8921	7910.0288	
213	22.8402	27.3822	12.9918	8125.2656	
243	22.9639	27.4287	12.9974	8186.6713	
273	23.0141	27.2799	12.9125	8106.7561	
303	23.3027	27.3710	12.9694	8272.1194	
333	23.4940	27.2233	12.9308	8270.3355	
363	23.8110	27.0839	13.0030	8385.5663	
393	24.2660	26.7198	12.8652	8341.5727	

Supplementary Table 4. Thermal expansion coefficients for compound **1o** in a cooling and heating cycle between 408 and 123 K.

T(K)	α_a(× 10^{-6} K^{-1})	α_b (× 10^{-6} K^{-1})	α_c(× 10^{-6} K^{-1})	β_v(× 10^{-6} K^{-1})
123				
153	238			669
173	128			166
183	125			56
213	213			340
223	187			222
243	206			320
273	180			189
278	210			291
303	221			273
328	213			201
333	230			234
363	260	-171		265
378	281	-255	-422	232
393	307	-308	-353	215
408	394	-469	-230	241

All α_a and β_v values were calculated relative to the parameters at 123 K. Therefore, the coefficient at each temperature T_i represents the thermal expansion over the temperature range from 123 K to T_i. All α_b and α_c values were calculated relative to the parameters at 333 K and 363 K, respectively.

Supplementary Table 5. Thermal expansion coefficients for compound **1o** between 333 and 408 K.

T(K)	α_a(× 10^{-6} K^{-1})	α_b (× 10^{-6} K^{-1})	α_c(× 10^{-6} K^{-1})	β_v(× 10^{-6} K^{-1})
333				
363	450	-171		464
378	494	-255	-422	215
393	548	-308	-353	143
408	814	-469	-230	251

All α_a, α_b and β_v values were calculated relative to the parameters at 333 K. All α_c values were calculated relative to the parameters at 363 K.

Supplementary Table 6. Comparison of thermal expansion coefficients for selected materials.

Compound	*T* (K)	α_{PTE} (× 10^{-6} K^{-1})	α_{NTE} (× 10^{-6} K^{-1})	Reference
$Ag_3Co(CN)_6$	10~500	132	−130	1
ZrW_2O_8	0.3~1050	NA	−9.1	2
[Ag(en)]NO_3-I	120~360	149	−90	3
Cd(im)	100~300	93	−23	4
Ag(mim)	20~300	130	−25	5
[Zn(OH)(niba)]	100~370	137	NA	6
[Zn(OH)(niba)]·MeOH	100~370	166	NA	6
[Zn(OH)(niba)]·*i*-PrOH	100~370	76	NA	6
HMOF-1	100~380	177	NA	7
FMOF-1	90~295	230	−170	8
MCF-18	119~295	81	NA	9
MCF-18·DMF	119~295	12~242	NA	9
MCF-18·MeOH	119~295	81~437	NA	9
MCF-34	127~673	224	−107	10
MCF-34·DMF	127~445	152 or 237	−56 or −116	10
MCF-82	112~300	482	−218	11
(S,S)-Octa-3,5-diyn-2,7-dio	225~330	156~515	−85	12
2(4PazP)·(4,6-diCl res)	260~290	316	−116	13
aspirin	93~323	93	NA	14
PHA-*α*	223~348	260	−80	15
Compound **1o**	123~408	125~394	NA	This work
Compound **1o**	333~408	450~814	−171 ~ −469	This work

Supplementary Table 7. Structural parameters *φ*, *θ*, *η*, *δ*, and *κ* for compound**1o** during a heating and cooling cycle from 123 to 438 K and then back to 123 K.

T(K)	*φ*	*θ*	*η*	*δ*	*κ*	
123	53.68	85.2	165.1	70.9	18.1	heating
153	53.88	86.2	164.1	71.8	19.4	
183	54.26	86.2	164.8	71.8	19.1	
213	54.15	89.2	163.4	74.6	19.5	
243	54.20	89.0	164.0	74.5	19.9	
273	52.24	90.6	163.5	76.8	20.1	
303	52.97	93.0	162.2	79.0	22.2	
333	52.00	95.4	161.2	82.4	23.6	
363	51.28	100.3	158.9	91.7	26.5	
393	49.52	107.2	156.0	95.6	30.9	
438	45	117.9	117.9	107.7	107.7	
303	45					cooling
123	45					

References for *Table S6*:

1 Goodwin, A. L., Calleja, M., Conterio, M. J., Dove, M. T., Evans, J. S. O., Keen, D. A., Peters, L. & Tucker, M. G. Colossal positive and negative thermal expansion in the framework material $Ag_3[Co(CN)_6]$. *Science* **319**, 794−797 (2008).

2 Mary, T. A., Evans, J. S. O., Vogt, T. & Sleight, A. W. Negative thermal expansion from 0.3 to 1050 Kelvin in ZrW_2O_8. *Science* **272**, 90−92 (1996).

3 Cai, W. & Katrusiak, A. Giant negative linear compression positively coupled to massive thermal expansion in a metal-organic framework. *Nat. Commun.* **5**, 4811 (2014).

4 Collings, I. E., Cairns, A. B., Thompson, A. L., Parker, J. E., Tang, C. C., Tucker, M. G., Catafesta, J., Levelut, C., Haines, J., Dmitriev, V., Pattison, P., & Goodwin, A. L. Homologous critical behavior in the molecular framework $Zn(CN)_2$ and Cd(imidazolate)$_2$ *J. Am. Chem. Soc.* **135**, 7610−7620 (2013).

5 Ogborn, J. M., Collings, I. E., Moggach, S. A., Thompson, A. L. & Goodwin, A. L. Supramolecular mechanics in a metal-organic framework. *Chem. Sci.* **3**, 3011−3017 (2012).

6 Grobler, I., Smith, V. J., Bhatt, P. M., Herbert, S. A. & Barbour, L. J. Turnable anisotropic thermal expansion of a porous zinc(II) metal-organic framework. *J. Am. Chem. Soc.* **135**, 6411−6414 (2013).

7 DeVries, l. D., Barron, P. M., Hurley, E. P., Hu, C. H. & Choe, W. ″Nanoscale lattice fence″ in a metal-organic framework: interplay between hinged topology and highly anisotropic thermal response. *J. Am. Chem. Soc.* **133**, 14848−14851 (2011).

8 Yang, C., Wang, X. P. & Omary, M. A. Crystallographic observation of dynamic gas adsorption sites and thermal expansion in a breathable fluorous metal–organic framework. *Angew. Chem. Int. Ed.* **48**, 2500−2505 (2009).

9 Wei, Y. S., Chen, K. J., Liao, P. Q., Zhu, B. Y., Lin, R. B., Zhou, H. L., Wang, B. Y., Xue, W., Zhang, J. P. & Chen, X. M. Turning on the flexibility of isoreticular porous coordination frameworks for drastically tunable framework breathing and thermal expansion. *Chem. Sci.* **4**, 1539−1546 (2013).

10 Zhou, H. L., Lin, R. B, He, C. T., Zhang, Y. B., Feng, N. D., Wang, Q., Deng, F., Zhang, J. P. & Chen, X. M. Direct visualization of a guest-triggered crystal deformation based on a flexible ultramicroporous framework. *Nat. Commun.* **4**, 2534 (2013).

11 Zhou, H. L., Zhang, Y. B., Zhang, J. P. & Chen, X. M. Supramolecular-jack-like guest in ultramicroporous crystal for exceptional thermal expansion behavior. *Nat. Commun.* **6**, 6917 (2015).

12 Das, D., Jacobs, T. & Barbour, L. J. Exceptionally large positive and negative anisotropic thermal expansion of an organic crystalline material. *Nat. Mater.* **9**, 36−39 (2010).

13 Hutchins, K. M., Groeneman, R. H., Reinheimer, E. W., Swenson, D. C. & MacGillivray, L. R. Achieving dynamic behaviour and thermal expansion in the organic solid state via co-crystallization. *Chem. Sci.* **6**, 4717−4722 (2015).

14 Bauer, J. D., Haussühl, E., Winkler, B. & Arbeck, D. Elastic properties, thermal expansion, and polymorphism of acetylsalicylic acid. *Cryst. Growth. Des.* **10**, 3132−3140 (2010).

15 Panda, M. K., Runčevski, T., Chandra Sahoo, S., Belik, A. A., Nath, N. K., Dinnebier, R. E., & Naumov, P. Colossal positive and negative thermal expansion and thermosalient effect in a pentamorphic organomettalic martensite. *Nat. Commun.* **5**, 4811 (2014).
